# Testing Phylogenetic Placement Accuracy of DNA Barcode Sequences on a Fish Backbone Tree: Implications of Backbone Tree Completeness and Species Representation

**DOI:** 10.1002/ece3.70817

**Published:** 2025-01-07

**Authors:** M. A. Thanuja M. Fernando, Jinzhong Fu, Sarah J. Adamowicz

**Affiliations:** ^1^ Department of Integrative Biology University of Guelph Guelph Ontario Canada

**Keywords:** analytical tools, DNA barcodes, phylogenetic accuracy, phylogenetic placement analysis, stratified sampling method

## Abstract

Advancements in DNA sequencing technology have facilitated the generation of a vast number of DNA sequences, posing opportunities and challenges for constructing large phylogenetic trees. DNA barcode sequences, particularly COI, represent extensive orthologous sequences suitable for phylogenetic analysis. Phylogenetic placement analysis offers a promising method to integrate COI data into tree‐building efforts, yet the impacts of backbone tree completeness and species composition remain under‐explored. Using a dataset comprising 27 genes and 4520 species of bony fishes, we assessed the accuracy of phylogenetic inference by “placing” COI sequences onto backbone trees. The backbone tree completeness was varied by subsampling 20%, 40%, 60%, 80%, and 99% of the total species separately, followed by placement of those missing species based on their COI sequences using software packages EPA‐ng and APPLES. We also compared the effects of biased, random, and stratified sampling strategies; the latter ensured the representation of all major lineages (Family) of bony fish. Our findings indicate that the placement accuracy is consistently high across all levels of backbone tree completeness, where 70%–78% missing species are correctly placed (by EPA‐ng) in the same locations as the reference tree derived from the complete data. High completeness produces slightly high placement accuracy, although in many cases the differences are nonsignificant. For example, at the 99% completeness level with stratified sampling, EPA‐ng placed 78% missing species correctly, and when only considering placement with high confidence (LWR > 0.9), the percentage is 87%. Additionally, stratified sampling outperforms random sampling in most cases, and biased sampling has the worst performance. The likelihood‐based EPA‐ng consistently provide higher accurate placements than the distance‐based APPLES. In conclusion, COI‐based placement analysis represents a potential route of using the available vast barcoding data for building large phylogenetic trees.

## Graphical abstract

This study evaluates the accuracy of incorporating COI DNA barcode sequences into phylogenetic trees of bony fishes, utilizing a dataset of 27 genes across 4520 species. Our findings indicate that the placement accuracy is consistently high across all levels of backbone tree completeness, and that stratified sampling consistently outperforms random sampling in most cases. The likelihood‐based EPA‐ng consistently provide higher accurate placements than the distance‐based APPLES. In conclusion, COI‐based placement analysis represents a potential route of using the available vast barcoding data for building large phylogenetic trees.

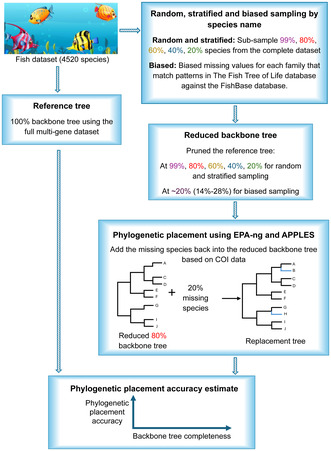

## 1 Introduction

Reconstructing the history of life is a central pillar in modern evolutionary biology, and the availability of massive DNA sequence data makes building large phylogenetic trees possible. Large phylogenetic trees that include thousands of species (tips) have provided many novel fundamental functions in biology (Zhu et al. 2019; Zuntini et al. 2024). For instance, they not only reveal large evolutionary patterns (e.g., Rabosky et al. 2018; Zuntini et al. 2024), but these patterns also help to set research and conservation priorities (e.g., Jetz and Pyron 2018), resolve critical phylogenetic relationships of large and diverse groups (e.g., Zaher et al. 2019; Zhu et al. 2019), and evaluate phylogenetic diversity and community structures (e.g., Lum, Rheindt, and Chisholm 2022; Staab et al. 2021). Data availability has greatly increased in the last three decades with the rapid advances in DNA sequencing technologies and the establishment of many public databases (e.g., GenBank). However, analyzing large amounts of data remains computationally challenging, and there is a high demand for analytical tools for building large phylogenetic trees (Park, Zaharias, and Warnow 2021; Zaharias and Warnow 2022). Existing tools often struggle with large datasets, resulting in inefficiencies, system failures, or an inability to process data altogether. Furthermore, the available DNA sequence data are highly variable, including various genes, various sequence lengths, and various methods of obtaining the sequences. How to incorporate these heterogeneous data is another challenging task (Roch and Steel 2015; Sayyari, Whitfield, and Mirarab 2017; Smirnov and Warnow 2021; Zaharias and Warnow 2022).

Phylogenetic placement is an efficient alternative approach for inferring large phylogenies (Balaban, Sarmashghi, and Mirarab 2020; Barbera et al. 2019; Czech et al. 2022). It expands an existing reference tree (“backbone tree”) by identifying the most favorable positions for a few newly acquired query sequences and placing them on the backbone tree, rather than constructs an entire phylogenetic tree (Balaban, Sarmashghi, and Mirarab 2020). This approach is mostly used for placing an unknown sequence into a taxonomic group (e.g., a species; Matsen, Kodner, and Armbrust 2010; Barbera et al. 2019) and has been extended to inferring large phylogenies (Wedell, Cai, and Warnow 2023). There are several advantages of placement analysis over *de novo* reconstruction of a full phylogenetic tree (Balaban, Sarmashghi, and Mirarab 2020; Czech et al. 2022). First, it is computationally efficient. Each query sequence is independently placed and computation can be readily parallelized (Matsen, Kodner, and Armbrust 2010). Also, placement avoids costly computation steps such as alignment and searching for the best whole tree (Linard, Swenson, and Pardi 2019; Turakhia et al. 2021). Second, placement is more scalable than *de novo* reconstruction. Relationships among the query sequences are not evaluated, avoiding to investigate an exponential number of phylogenetic hypotheses (Matsen, Kodner, and Armbrust 2010). This scalability allows us to deal with very large trees and to address dynamic scenarios where novel sequences are regularly generated (Balaban, Sarmashghi, and Mirarab 2020; Koning, Phillips, and Warnow 2021). Third, it performs well to accommodate sequence length heterogeneity (Zaharias and Warnow 2022). While the sequences used to create the backbone tree can be long and from multiple genes, a short query sequence can be easily placed on the backbone tree with placement analysis (Blanke and Morgenstern 2021).

There are at least four classes of phylogenetic placement methods (Zaharias and Warnow 2022). Maximum likelihood‐based (ML) methods are generally more accurate but computationally less efficient than others (Balaban, Sarmashghi, and Mirarab 2020; Linard, Swenson, and Pardi 2019). Several software packages, such as RAxML‐EPA and EPA‐ng, implement ML methods. On the other hand, parsimony‐based methods, implemented in software such as UShER (Turakhia et al. 2021), distance‐based methods, implemented in software such as APPLES (Balaban, Sarmashghi, and Mirarab 2020), and k‐mer‐based methods, implemented in software such as RAPPAS (Linard, Swenson, and Pardi 2019) are computationally efficient, but often less accurate. Additionally, different software packages also have their advantages and limitations (Balaban, Sarmashghi, and Mirarab 2020; Barbera et al. 2019). For example, RAxML‐EPA (Berger, Krompass, and Stamatakis 2011) and EPA‐ng (Barbera et al. 2019) are optimized for batch processing placement sequences (as opposed to one at a time). APPLES is also an efficient and accurate placement tool (Balaban, Sarmashghi, and Mirarab 2020).

The availability of massive DNA barcoding sequences provides an opportunity for a significant expansion of the current animal phylogenies using phylogenetic placement analysis. DNA barcoding sequences refer to sequences from a short fragment (~670 bp) of mitochondrial gene cytochrome c oxidase subunit 1 (COI), which have been used primarily to identify species and are deposited in the Barcoding of Life Database (BOLD; Cheng et al. 2023; Hebert, Ratnasingham, and DeWaard 2003; Ratnasingham and Hebert 2007). These data have several advantages over other DNA sequences. First, the DNA barcoding sequences represent the largest homologous DNA sequence data. BOLD currently holds 20.1 million sequences and 25.5 million specimen records representing at least 359,322 species (queried on December 05, 2024). Second, these data are generated using standardized protocols with high‐quality control (Gostel and Kress 2022; Paz and Rinkevich 2021). Third, all sequences are accompanied by reference voucher specimens that ensure the accuracy, reliability, and longevity of biodiversity data (Meiklejohn, Damaso, and Robertson 2019; Ratnasingham et al. 2024). COI sequences by themselves may not be very powerful for *de novo* phylogenetic construction due to their short length; however, they can be used for phylogenetic placement analysis. Considering the large number of available COI sequences from the BOLD database, using COI to fill the tips of a backbone tree, which can be based on multiple gene sequences, may represent a powerful approach to build large phylogenetic trees (Chesters et al. 2023; Frandsen et al. 2016).

The accuracy of placement analysis relies on the accuracy of the backbone trees (Balaban, Sarmashghi, and Mirarab 2020; Barbera et al. 2019; Matsen, Kodner, and Armbrust 2010). One factor, the completeness (or density) of species sampling for the backbone tree, is expected to have a significant impact on the accuracy of phylogenetic placement (Balaban, Sarmashghi, and Mirarab 2020). Additionally, appropriate representation of the major lineages on the backbone tree is likely critical. Representative species from major groups (e.g., Phylum; Mahé et al. 2017) are often sought for placement analysis; however, there are various biases in selecting species for constructing backbone trees in reality. For example, data are generally more available for common or economically important species than obscure species, and species selection is often biased toward those species (Liu et al. 2024; Radosavljević et al. 2023). While both issues are well‐recognized, neither has been explicitly tested.

The ray‐finned fishes, the Class Actinopterygii, provide an excellent study system to test the approach of building large phylogenetic trees by placing DNA barcode sequences on multigene backbone trees. The group currently has 35,600 species (FishBase; Froese and Pauly 2021), and Rabosky et al. (2018) provides a well‐sampled ray‐finned fish backbone tree. They gathered DNA sequence data from 27 genes representing 11,638 species and constructed a tree using maximum likelihood analysis. Furthermore, 6796 of these species have COI DNA barcode sequences. The large dataset allows us to manipulate the data to address our specific questions related to backbone tree completeness and lineage representation.

Our primary objective is to test the accuracy of phylogenetic placement of DNA barcoding (COI) sequences on a multigene backbone tree, with different levels of species completeness and variations of representation. More specifically, we use the dataset and phylogeny from Rabosky et al. (2018), and subsample their dataset to create different levels of backbone tree completeness and different species representations. We first test how the level of species completeness of the backbone tree influences measures of phylogenetic placement accuracy. Second, we evaluate the influence of species sampling patterns for the backbone tree on phylogenetic placement accuracy, including random sampling, taxonomically stratified sampling, and biased sampling. Third, we compare two phylogenetic placement software tools, the likelihood‐based EPA‐ng and distance‐based APPLES in terms of phylogenetic accuracy and computational efficiency. Our results will provide a foundation for various strategies that researchers may choose and a potentially powerful approach for using DNA barcoding sequences to build large phylogenetic trees.

## 2 Materials and Methods

### 2.1 Data Acquisition and Quality Control

DNA sequence data for building the backbone trees were retrieved from “The Fish Tree of Life” ray‐finned fishes dataset, which consists of 11,638 species and DNA sequence data from 27 genes (Rabosky et al. 2018). Our initial quality evaluation detected several erroneous sequences in the dataset (e.g., *Silurus asotus* COI sequence appears to be a bacterial contamination *Pseudoalteromonas* sp.) as well as alignment issues. Consequently, we filtered the data by removing (1) sequences with poor alignment or misalignment in positions that are generally conserved, such as *Siganus canaliculatus* (COI gene), *Citharichthys sordidus* (CytB gene), *Citharichthys stigmaeus* (CytB gene), *Citharichthys sordidus* (COI gene), *Citharichthys stigmaeus* (COI gene), (2) sequences with large gaps (> 150 bp), such as *Callogobius bifasciatus* (COI) and *Siganus canaliculatus* (COI) in its 5′ end, *Acanthurus olivaceus* (Rag‐1 gene), *Acanthemblemaria rivasi* (Rag‐1 gene), and (3) short gene sequences < 100 bp (e.g., ND4 gene of *Salmo trutta* is only 70 bp long). Lastly, to make a robust backbone tree, we removed species with data from only one or two genes and retained species with data from a minimum of three genes, and one of them must be COI, which allowed us to test placement by the COI sequences. To resolve alignment issues, the multigene sequence dataset was split into 27 single‐gene datasets and each dataset was subjected to realignment using AlignSeqs() in the DECIPHER package. The 12S and 16S genes, as part of the ribosomal RNA family with well‐defined secondary structures crucial for their function, were aligned using structural information to improve accuracy. Consequently, the “useStructures” parameter in AlignSeqs() was set to TRUE for the 12S and 16S genes, while default parameters were applied to the other 25 genes. Additionally, the RNAStringSet argument was used for the 12S and 16S datasets, while the DNAStringSet parameter was employed for the remaining genes. The realigned data were concatenated to generate a multigene sequence dataset, which was used for all subsequent analysis. As a result, 4520 species were retained in the dataset and each species possessed data from a minimum of three genes. The total alignment length was 24,809 base pairs (bp). Due to missing genes and gaps, sequence lengths varied from 1305 to 17,497 bps excluding gaps, with an average of 4880 bps. We used this dataset as our “reference dataset.”

### 2.2 Phylogenetic Construction of Reference Tree and Reduced Backbone Trees

We first constructed a reference tree for the 4520 species based on our reference dataset. A maximum likelihood method implemented in RAxML version 8.2.12 (Stamatakis 2014) was used. For our phylogenetic analyses, we employed the General Time Reversible (GTR) model combined with the Category (CAT) model to account for nucleotide substitution and evolutionary rate heterogeneity. The GTRCAT model was selected because of its demonstrated high performance and reduced computational time, making it particularly suitable for large‐scale datasets (Stamatakis 2015). We used the same tree root as that of Rabosky et al. (2018) and did not include an outgroup to simplify the subsequent subsampling process.

**FIGURE 1:**
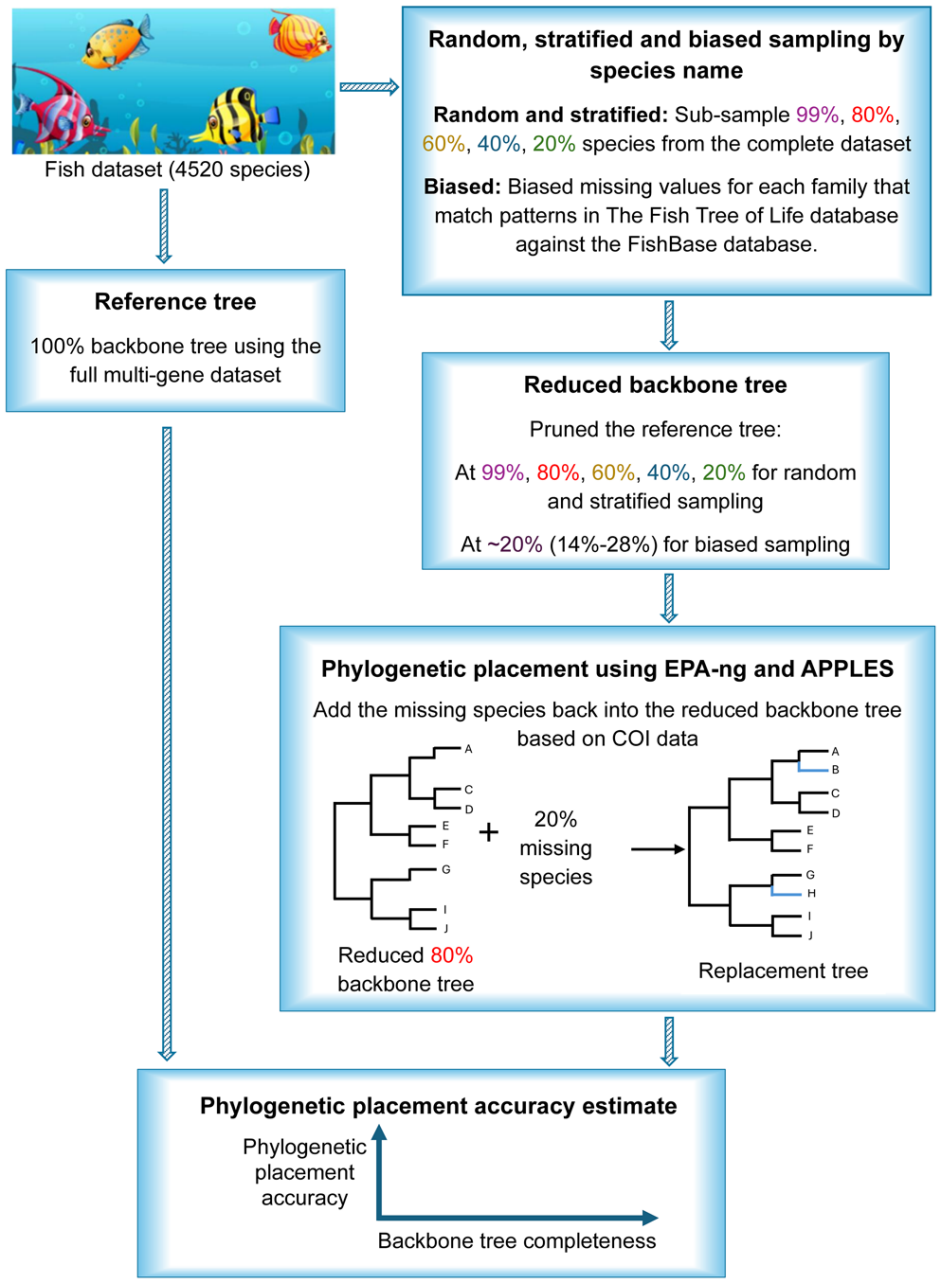
Workflow of phylogenetic placement accuracy analysis.

To test the impact of backbone tree completeness on placement accuracy, we subsampled the 4520 species to create datasets representing 20%, 40%, 60%, 80%, and 99% of the total species. Based on the missing species (species that were not subsampled) from these datasets, we pruned the reference tree to generate reduced backbone trees at each level using the drop. tip() function in the R package *ape* version 5.8 (Paradis and Schliep 2019), which served as the basis for the phylogenetic placement analysis (Figure 1).

To focus on assessing the impact of backbone completeness and minimizing other noises, such as species identity, the sampling process started at 99% followed by 80%. The 80% samples were extracted from the 99% samples by removing 19%. Likewise, the 60% samples were extracted from the 80% samples, the 40% samples were extracted from the 60% samples, and the 20% samples were extracted from the 40% samples. The entire process was repeated 10 times, and each sampling cycle was performed independently. A workflow chart is presented in Figure 1.

Three sampling strategies were applied: random, stratified, and biased. For random sampling, an N% (e.g., 20%, 40%) of species were drawn at random from the 4520 species without considering their taxonomic identifications. Random sampling may result in some major groups being entirely omitted. The stratified sampling approach first divided all species into smaller groups, or strata, and sampled N% of species from each stratum. We used the taxonomic rank, Family, as our stratum, and sampled N% of species from each fish family and retained all families in all reduced reference datasets. For species‐poor families (e.g., families with fewer than 5 species), all species were retained at each level of sampling.

Biased sampling represents a scenario where certain groups (e.g., economically important groups, groups that inhabit easily accessible habitats) are better studied than others. We empirically generated the missing percentage values for each family that match patterns in The Fish Tree of Life database against the FishBase database (Froese and Pauly 2021). We used the FishBase to represent the “complete” list of fish and The Fish Tree of Life database to represent the “biased” sample and counted the number of species that were missing from the latter for each family. We then applied the estimated missing percentage values to the reference dataset to generate the reduced reference datasets.

### 2.3 Phylogenetic Placement Analysis

Using placement analysis, we placed all of the missing species (species that were not subsampled) back on the reduced backbone trees based on their COI gene sequences to produce the placement trees, which would have the same species composition as the reference tree. The reduced backbone trees, the reduced reference datasets, and the COI sequences of the missing species (query sequences) were input data for the phylogenetic placement analysis.

We used two software packages for placement analysis, EPA‐ng version 0.3.4 (Barbera et al. 2019) and APPLES version 2.0.11 (Balaban, Sarmashghi, and Mirarab 2020; Balaban et al. 2021). EPA‐ng uses a likelihood‐based method, which is generally considered the most accurate (Barbera et al. 2019). It is the newer version of RAxML‐EPA (Berger, Krompass, and Stamatakis 2011; Stamatakis 2014), modified for speed and capacity (Barbera et al. 2019). APPLES uses a distance‐based method, which generally offers fast, scalable, and accurate phylogenetic placement for large datasets (Balaban, Sarmashghi, and Mirarab 2020; Balaban et al. 2021). For EPA‐ng, COI sequences were first aligned using MAFFT (Katoh et al. 2002). The COI alignment and the reduced multigene reference datasets were input into the program in fasta format. COI short read sequences were required to undergo alignment against the multigene reference dataset. Subsequently, the multigene alignment was partitioned into distinct sets to facilitate alignment with the COI sequences. The (pruned) reduced backbone tree in Newick format was inputted using the “‐tree” command and specified the model as GTR in EPA‐ng for placing query sequences onto the fixed backbone tree. For example, when we used an 80% backbone tree, the reduced reference dataset consisted of the 80% multigene sequences while the 20% missing species consisted of COI short read sequences, which we placed on the backbone tree. The APPLES tool requires that the query sequences have the same length as the reference sequences, and therefore, we used COI sequences as reference (instead of the multigene sequences). The placement analysis was repeatedly applied to the ten replicates generated at the sampling stage for different levels of sampling completeness in the backbone trees (99%, 80%, 60%, 40%, 20%). To facilitate the downstream analysis, we converted the output tree file of the placement analysis (e.g., jplace file) to labeled Newick files using the Genesis v0.30.0 library (Czech, Barbera, and Stamatakis 2020). These placement trees were compared to the reference tree for accuracy estimates.

### 2.4 Phylogenetic Accuracy Estimates

We used the percentage of correct placements (PCP) metric to measure the accuracy of the phylogenetic placement. The reference tree (derived from the 4520 species and 27‐gene sequence alignments) was used to represent the “correct” tree. The PCP was estimated by comparing the sister clade of a placed species on the placement tree to the sister clades on the reference tree. For each placed species, we first identified the sister group of the species from the placement tree. We then identified the most inclusive clade from the reference tree that included all members of the species' sister group from the placement tree. By comparing the members of the most inclusive clade from the reference tree and the sister group from the placement tree, we obtained a species list representing their membership difference. If all species on the difference list were from the “missing species” (those not sampled on the reduced backbone tree), the placement was considered correct. If any of the different species was found on the reduced backbone tree, the placement was deemed incorrect. Furthermore, we identified species with high placement confidence (e.g., likelihood weight ratio > 0.90) and estimated their PCP. We expected that placements with high confidence would likely have a high correct rate. Software tool APPLES does not provide a confidence estimate, and therefore, we did not conduct this analysis for APPLES.

### 2.5 Statistical Analysis

We compared the PCP metric of phylogenetic accuracy (response variable) to test if the sampling completeness (first predictor variable) depend on the sampling type (second predictor variable). The repeated measures approach was selected due to the sequential data‐dropping technique, where the species included in each level of sampling completeness were not random within each sampling run. We first assessed whether the data conformed to the assumptions of parametric tests. Outliers were checked for significance within each cell of the design, and normality was tested using the Shapiro–Wilk test. Since our data violated the assumptions of parametric tests, we used a nonparametric aligned rank transform (ART) ANOVA test. This analysis was conducted using the ANOVA function in the R package *car* version 1.0‐9 (Fox and Weisberg 2019) with ART‐transformed data from the R package *ARTool* version 0.11.1 (Elkin et al. 2021). To compare the accuracy between sampling strategies and placement tools, post hoc pairwise comparisons were performed using the contrast test (ART‐C) with the art.con function in *ARTool*. An alpha level of 0.05 was used for all statistical tests.

## 3 Results

The reference tree of 4520 species is available on GitHub and a summary tree is presented in Figure S1. We used the same tree root as Rabosky et al. (2018), which is between the family Polypteridae and the rest of the fish species. Overall, our tree was largely consistent with that of Rabosky et al. (2018).

**FIGURE 2:**
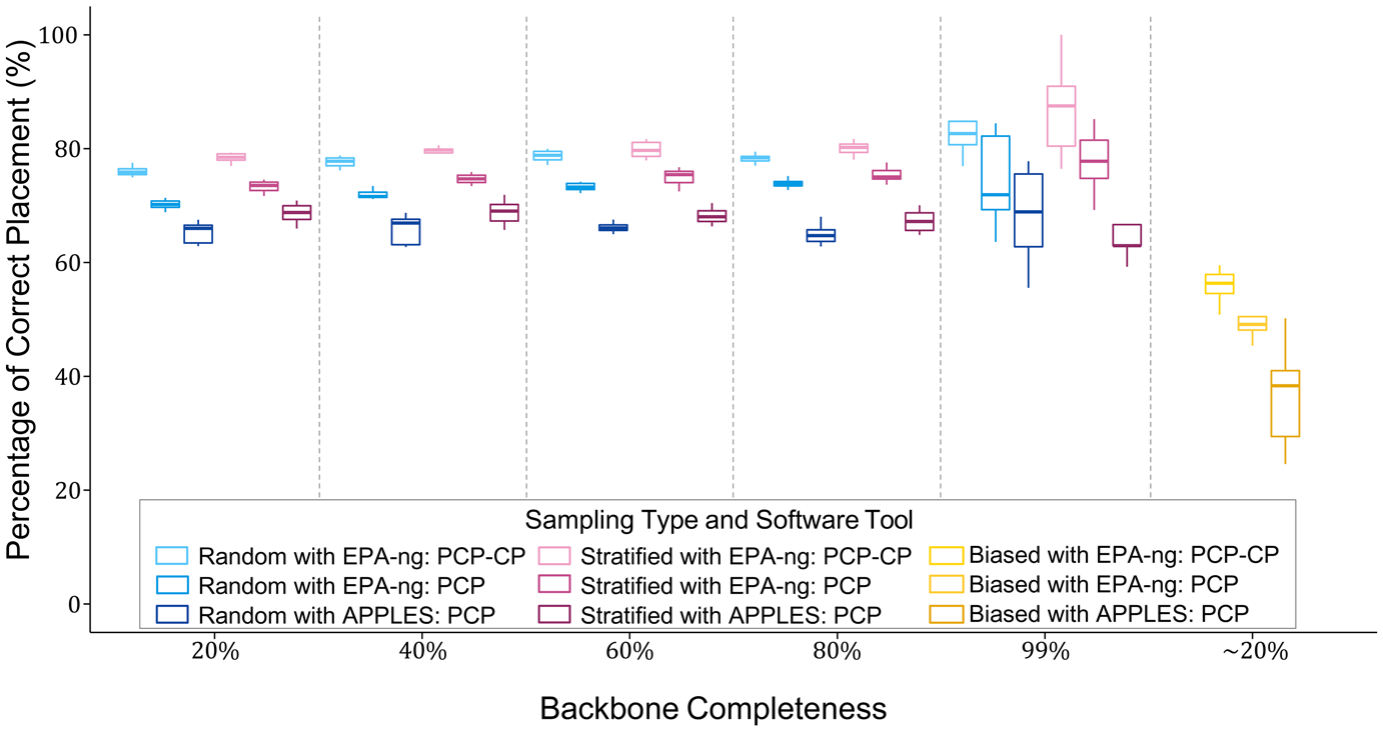
Boxplot of the percentage of correct placement (PCP) for random, stratified, and biased sampling strategies at five backbone tree completeness levels using EPA‐ng and APPLES. The biased sampling strategy use empirically generated levels of completeness (14%–28%), with an average approximately 20%.

The percentage of correct placements (PCP) results from EPA‐ng and APPLES are presented in Table 1 and Figure 2. For EPA‐ng, average PCP values generally varied from 70% to 78% for random and stratified sampling at all levels of backbone tree completeness. Placements with high confidence (LWR > 0.90) amounted to 77%–82% of all placements, and among them, PCP‐CP values varied between 76% and 87% (Table 1). There were several trends in the data. First, as expected, placements with high confidence had higher accuracy rate; PCP‐CP values were substantially higher than PCP values in all cases (Table 1, Figure 2). Second, stratified sampling always had higher PCP and PCP‐CP values than random sampling; however, in approximately half the cases, the differences were minor and statistically nonsignificant (Tables 1 and 2). Particularly at 99% completeness level, the differences were always nonsignificant (Table 2). Third, PCP values increased slightly along with the increase of backbone tree completeness for random sampling (Table 1). For example, both PCP and PCP‐CP values at 60%, 80%, and 99% completeness levels were higher than those at 20% level; PCP‐CP value at 99% level was higher than those at 40%, 60%, 80% levels. All other pairwise comparisons were nonsignificant (Table S1). For stratified sampling, however, the only significant different cases were that PCP‐CP values at 80% and 99% levels were higher than that at 20%, and all other pairwise comparisons were nonsignificant (Table S1). Fourth, EPA‐ng produced substantially higher PCP values in all cases than APPLES did (Table 1). Finally, with only an average of 57% (range 51%–67%) correct placements, the biased sampling was substantially worse than the stratified and random sampling (Figure 2). The biased sampling represented an approximately 20% completeness level.

**TABLE 1:** Placement accuracy estimates. Results from two sampling strategies at five backbone tree completeness levels using two software tools are presented. PCP = percentage of correct placements. CP = percentage confident placements where likelihood weight ratio, LWR > 0.9. APPLES does not provide a confidence estimate. PCP‐CP = percentage of correct placements among confident placements. Numbers are average from 10 iterations with ranges in parentheses.

Backbone completeness level	Random sampling				Stratified sampling			
	EPA‐ng			APPLES	EPA‐ng			APPLES
	PCP	CP	PCP‐CP	PCP	PCP	CP	PCP‐CP	PCP
20%	70 (69–71)	78 (76–80)	76 (75–78)	65 (58–68)	73 (72–75)	80 (78–81)	78 (77–79)	69 (66–71)
40%	72 (71–74)	79 (78–81)	78 (76–79)	66 (63–69)	75 (73–76)	78 (54–82)	80 (77–81)	67 (47–72)
60%	73 (72–74)	79 (78–81)	79 (77–80)	66 (62–68)	75 (73–77)	80 (79–81)	80 (78–82)	68 (66–70)
80%	74 (72–76)	80 (78–83)	78 (76–80)	65 (63–68)	75 (74–78)	78 (76–81)	80 (78–82)	67 (65–70)
99%	75 (64–84)	82 (76–89)	85 (77–97)	69 (56–78)	78 (69–85)	77 (63–93)	87 (77–100)	66 (59–82)

**TABLE 2:** *p* Values of pairwise comparisons of the percentage of correct placement (PCP) accuracy metric between random and stratified sampling strategies using EPA‐ng and APPLES. A nonparametric ART‐C (Contrast test) test is used due to violation of parametric assumptions. Bold = significant at 0.05 level.

Software tool	Backbone tree completeness				
	20%	40%	60%	80%	99%
EPA‐ng: PCP‐CP	**0.0133**	**0.0119**	0.646	**0.0323**	1.00
EPA‐ng: PCP	**0.0201**	**0.00564**	0.335	0.635	0.150
APPLES: PCP	**0.0497**	0.651	0.263	0.713	0.747

The average PCP values from APPLES ranged from 65% to 69% (Table 1, Figure 2). There was little trend in the data, and neither backbone tree completeness nor sampling strategies had any significant impact on PCP values (Table 2, Table S1), except that the biased sampling had a substantially lower PCP (average 37%, range 25%–51%) than other strategies.

## 4 Discussion

Our results clearly demonstrate that the placement analysis consistently places ~70%–87% of COI sequences in their “correct” locations on a backbone tree, even if its completeness is as low as 20%. Also, a backbone tree that includes representatives from all families (stratified sampling) generally results in more accurate placements. Furthermore, likelihood‐based EPA‐ng performs significantly better than distance‐based APPLES.

The placement analysis maintains a consistent and high level of accuracy (e.g., 70%–87% for EPA‐ng) across all levels of backbone tree completeness. This is surprising and unexpected; the completeness of the backbone tree is not a key parameter to the accuracy of phylogenetic placement analysis. Although the results from EPA‐ng show improvements with increased completeness (particularly at the 99% level), the differences are generally minor (Figure 2). Furthermore, the percentages of placements with high confidence (LWR > 0.90) are also relative constant (76%–87%; Table 1) at all levels of completeness. The only exception is biased sampling. We generated the biased sampling by mirror a real world situation (FishTree vs. FishBase) and it approximates a 20% level of backbone tree completeness. In all cases, the placement accuracy of biased sampling is substantially lower than other sampling strategies (Figure 2). Nevertheless, even at 99% completeness level, more than 13% on average high‐confident placements are incorrect. The primary cause of these inaccurate placements is likely the short length of the COI sequences. The short length provides limited phylogenetic information, and hence limited resolution. Also, maternal inheritance and lack of recombination of mitochondrial DNA may lead to different evolutionary trajectories and potentially discordant genealogies (Ferreira and Rodriguez 2024; Perea et al. 2016; Toews and Brelsford 2012). Other processes, such as incomplete lineage sorting, hybridization events, and selection pressures can also cause biases of mitochondrial genes in phylogenetic analysis (Perea et al. 2016; Toews and Brelsford 2012). Overall, placement represents an accurate approach for expanding large phylogenetic trees. With the vast amount of sequences available in the Barcodes of Life Database, using placement analysis to combine a solid backbone tree and COI sequence is likely an effective way of tapping into the available resources.

The stratified sampling strategy of the backbone tree provides better placement accuracy than other strategies in most situations. Having representatives from major lineages on the backbone tree clearly improves the placement accuracy (Figure 2; Table 1). Stratified sampling is common and realistic, and many researchers are already practising such stratified sampling in many different ways. For example, Wang et al. (2023) demonstrated that stratified sampling improved the accuracy of phylogenetic diversity metrics in fish communities. Similarly, Arato and Fitch (2021) utilized stratified sampling to examine phylogenetic signals in bird vocalizations. Rosenberg and Kumar (2003) also highlighted the importance of taxon sampling in bioinformatics, noting that careful taxon selection, often involving stratified sampling, is critical for accurate phylogenetic analysis. On the other hand, biased sampling also represents a common and realistic practice, and its performance in phylogenetic placement analysis is the worst. Species with economic or scientific importance, common or attractive species, or species inhabiting accessible habitats often receive biased sampling. Intentionally increasing representation across major lineages and minimizing biased sampling for the backbone trees are essential in placement analysis.

There are several available placement tools, and we used two, the likelihood‐based EPA‐ng and the distance‐based APPLES. In terms of accuracy, EPA‐ng performs better than APPLES in all the cases (Table 1, Figure 2). In terms of computation efficiency, both the tools are very fast for placement analysis (~10 s to ~22 s). However, EPA‐ng requires substantial data preparation, in particular, an alignment matching the COI sequences to the multigene sequence requires adding many gaps. On the other hand, APPLES is notable for its minimal data preparation requirements, making it highly user‐friendly and efficient. It works directly with raw sequence data in FASTA format without alignment, providing that both query sequences and reference data are on the same scale. Its simplicity, combined with compatibility with widely used phylogenetic tools, makes APPLES exceptionally convenient for large‐scale phylogenetic placement tasks (Balaban, Sarmashghi, and Mirarab 2020; Smirnov and Warnow 2021).

There are several caveats in this study. One potential limitation is our modest sample size of sampling iterations (i.e., 10 replicates). Nevertheless, the results obtained are highly consistent, with low to moderate variability among the replicates; therefore, our findings should be considered credible even with the small sample size. Additionally, our fish dataset represents only a modest subset of the true fish diversity and is likely a biased sample. Therefore, the accuracy of the reference tree and the placement trees cannot be objectively assessed. Our accuracy measurements only measure the differences between them.

We would like to make three recommendations based on our results. First, using stratified sampling strategy for backbone tree is always preferred. In our case, we included representative species from each fish Family in the Class Actinopterigii on backbone trees. Similar strategy can be applied to other levels of taxonomy. Other means of identifying stratum, such as using a phylogenetic tree, can also be applied. Second, the software package EPA‐ng is preferred for placing short‐read sequences, even it requires additional data preparation time compared to APPLES. EPA‐ng provides a higher accuracy as well as a measurement for placement confidence (e.g., LWR); the latter can be used to further root out low‐confident placements that have high error rate. Third, even though the differences are small, high backbone tree completeness does yield better placement accuracy, particularly at 99% level. Overall, using placement analysis of barcoding sequences (COI) to build large trees has high accuracy, which is likely useful for many ecological inferences, such as diversity estimates and ecological association studies. With the availability of a huge amount of new DNA barcoding sequences, this approach is highly promising.

## Author Contributions

**M. A. Thanuja M. Fernando:** data curation (lead), formal analysis (lead), investigation (lead), methodology (lead), project administration (equal), resources (lead), software (lead), visualization (lead), writing – original draft (lead). **Jinzhong Fu:** project administration (equal), supervision (equal), writing – review and editing (lead). **Sarah J. Adamowicz:** conceptualization (lead), funding acquisition (lead), project administration (equal), supervision (equal).

## Conflicts of Interest

The authors declare no conflicts of interest.

## Benefit Sharing Statement

This study adheres to benefit‐sharing principles by providing open access to the research data and codes. These resources are intended to support the scientific community by enabling the replication of our study, fostering new research, and enhancing educational opportunities. By sharing these resources, we aim to maximize the impact of our research and contribute to the collective knowledge base, facilitating advancements in the field. Researchers and interested parties are encouraged to use the provided resources, which include the realigned sequences from the FishTree of Life dataset and the phylogenetic tree files, for further research and educational purposes.

## Supporting information


**Figure S1.** Summary tree of the fish reference tree.

**Table S1.** *p* Values of pairwise comparisons of the percentage of correct placement (PCP) accuracy metric between backbone tree completeness for random and stratified sampling strategies using EPA‐ng and APPLES. A nonparametric ART‐C (Contrast test) test is conducted due to violation of parametric assumptions.

## Data Availability

The codes and datasets generated and analyzed during this study are available on GitHub at https://github.com/Thanu92/Realignment. This repository includes the scripts used for data analysis and visualization, as well as the processed data files. The research data, including the realigned sequences from the Fish Tree of Life dataset after removing sequences with issues, and the phylogenetic tree files are also provided on GitHub at https://github.com/Thanu92/Realignment. The Order/family summary tree is presented in the Supporting Informations (Figure S1) section accompanying this manuscript.
