# Supplementary figures and images for: Testing Phylogenetic Placement Accuracy of DNA Barcode Sequences on a Fish Backbone Tree: Implications of Backbone Tree Completeness and Species Representation
Source: Ecol Evol. 2025 Jan 7;15(1):e70817. doi: 10.1002/ece3.70817 (PMC11706799; doi:10.1002/ece3.70817)

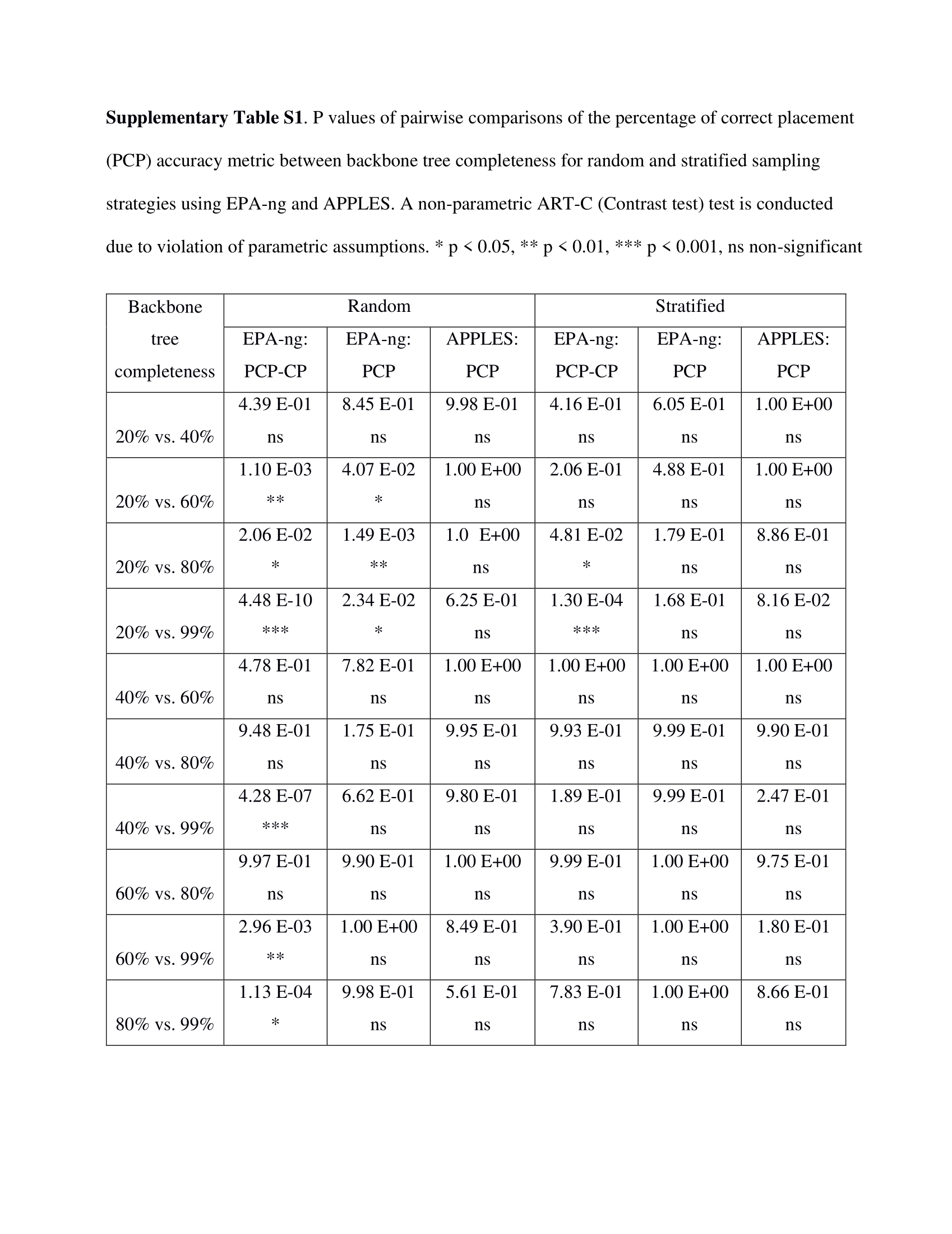

Supplement: Supplementary file 2 — Table S1. p Values of pairwise comparisons of the percentage of correct placement (PCP) accuracy metric between backbone tree completeness for random and stratified sampling strategies using EPA‐ng and APPLES. A nonparametric ART‐C (Contrast test) test is conducted due to violation of parametric assumptions. [file ECE3-15-e70817-s002.tiff]
